# Case Report: Decoding genetic risks of vascular parkinsonism: a case series

**DOI:** 10.3389/fgene.2025.1579454

**Published:** 2025-07-25

**Authors:** Ali Shalash, Salma El-Shafie, Peter George, Tamer Roushdy, Mai Fathy, Mohamed H. Yousef, Mahmoud El-Belkimy, Mohamed Ossama Abdulghani, Mohamed Salama

**Affiliations:** ^1^ Neurology Department, Faculty of Medicine, Ain Shams University, Cairo, Egypt; ^2^ Biology Department, The American University in Cairo, Cairo, Egypt; ^3^ Institute of Global Health and Human Ecology, The American University in Cairo, Cairo, Egypt

**Keywords:** vascular, parkinsonism, leukoencephalopathy, genetics, Egyptian

## Abstract

**Background:**

Vascular parkinsonism (VaP) is a subtype of parkinsonism which needs better characterization of its risks and determinants.

**Objective:**

The aim of this report is to present an understanding of genetic risks of vascular parkinsonism.

**Methods:**

Five participants diagnosed with VaP were recruited and Whole Exome Sequencing (WES) was performed to analyze deleterious variants in relevant genes associated with vascular and parkinsonian diseases.

**Results:**

We identified several candidate risk variants for VaP in our patients, particularly in LRRK2, PLA2G6, TGM6, BSN, UBR4, CD36 and NOTCH3, that are different from the classical Parkinson’s disease -associated variants.

**Conclusion:**

In this case series we highlighted the complexity of genetic contributions to VaP through predicted deleterious variants in genes associated with parkinsonism, cerebrovascular disease as well as collagen-related genes.

## Introduction

Vascular Parkinsonism (VaP) is a form of parkinsonism associated with cerebrovascular disease. It predominantly affects the elderly accounting for 4.4%–12% of all parkinsonism cases worldwide (George et al., 2024), This percentage was reported as high as 28.6% in an Egyptian study (El-Tallawy et al., 2013).

Clinically, VaP presents with lower body parkinsonism, impaired gait and cognitive dysfunction, accompanied by white matter lesions (WMLs) or lacunes found in brain neuroimaging (George et al., 2024). Three main subtypes of VaP exist: acute (post-stroke), insidious (more debatable and common with gradual onset and extensive WMLs), and mixed (pathologies from both vascular and neurodegenerative diseases) (Mostile et al., 2018).

Historically, the diagnosis of insidious VaP has been controversial, lacking a distinct structural imaging pattern and having poorly established correlations between neurological symptoms and imaging findings (George et al., 2024). The syndrome’s loose criteria lead to a highly variable clinical picture, often overlapping with neurodegenerative diseases like Parkinson’s Disease (PD) and Progressive Supranuclear Palsy (PSP) as well as non-neurodegenerative conditions such as cerebral autosomal dominant arteriopathy with subcortical infarcts and leukoencephalopathy (CADASIL) and Normal Pressure Hydrocephalus (NPH) (Mostile et al., 2018).

Genetic factors, particularly mutations associated with leukoencephalopathies, such as CSF1R, COL4A1/2, NOTCH3, NOTCH2NLC and COL22A1 have emerged as potential contributors to VaP clinical picture, proposing an alternative diagnosis and warranting investigation to clarify the genetic etiology of VaP (Marsili et al., 2023). “Adult leukoencephalopathy-associated parkinsonism” has been previously suggested as alternative term to the debatable term “insidious VaP” (George et al., 2024).

This study aims to explore the genetic pathophysiology of VaP through whole exome sequencing (WES), analyzing deleterious variants in relevant genes associated with vascular and parkinsonian diseases.

## Methods

The study involved five participants diagnosed with VaP according to Zijlmans criteria (Zijlmans et al., 2004). Patients with other diagnoses such as Parkinson’s disease (PD) and atypical parkinsonism were not included. The study was conducted in accordance with the Declaration of Helsinki, and the protocol was approved by the ethical committee of the Faculty of Medicine, Ain Shams University (George et al., 2024). All subjects gave their informed consent for inclusion before they participated in the study.

Patients were subjected to comprehensive clinical evaluations, including motor and cognitive assessments (George et al., 2024).

Whole Exome Sequencing (WES) was performed on DNA isolated from peripheral blood using Illumina Novaseq 6000 platform with a coverage of 100x. Variants with read depth <10 were filtered out. ANNOVAR was used for variant annotation (Wang, 2010) for filtering variants with a minor allele frequency (MAF ≤1%) according to 1000 Genomes or GnomAD and loss-of-function (LoF) or missense functional consequence according to RefSeq annotation with CADD score >20 (representing the most deleterious 1% of all amino acid substitutions) (Rentzsch et al., 2019) and consensus of deleteriousness by 2 or more tools provided by ANNOVAR dbnsfp47a database (for functional prediction of variants in whole-exome data). Variants within genes associated with parkinsonian or cerebrovascular disease were prioritized. For further assessment of the clinical relevance of the prioritized genes, the genes harboring the prioritized variants were checked for expression in brain tissue in GTEx (https://gtexportal.org/home/) and the Mouse Genome Informatics (MGI; https://www.informatics.jax.org/) to identify genes whose knockout demonstrated an abnormal neurological phenotype in mice. The use of the American College of Medical Genetics ACMG or Clinvar classification of pathogenicity was not assumed appropriate for genetic analysis of parkinsonism as it is intended for Mendelian single-gene disorders while mostly parkinsonism is sporadic and possibly multiple genetic risk factors are involved, however both annotations were reported for further insight. From this prioritized gene list, we chose to highlight genes, harboring deleterious variants, that were observed in 2 or more patients using a voting approach, allowing for allelic heterogeneity where different deleterious variants within same gene may cause disease or increase risk of disease (Chen et al., 2013).

The more comprehensive filtered gene list, not restricted to parkinsonism or cerebrovascular-associated genes, was used for enrichment analysis to investigate overrepresented pathways using enrichr (Chen et al., 2013). This analysis was done with the hypothesis in mind that pathways important for cerebrovascular function or PD/parkinsonism pathogenesis could be overrepresented by deleterious variants in their corresponding genes where the polygenic defects contribute to the disease. This was done for the whole filtered gene list as well as a subset of genes harboring the most deleterious variants, predicted using REVEL cut-off of 0.7, recommended for prioritizing pathogenic variants (Ioannidis et al., 2016; Hopkins et al., 2023).

## Results

Participants had a mean age of 71.8 ± 11.2 years. Notable vascular risk factors were present and there was no family history of PD. Approximately 80% did not significantly respond to the levodopa challenge test, and 80% showed insidious onset of symptoms. Cognitive impairment was observed across the cohort (Table 1).

**TABLE 1:** Clinical characteristics of VaP patients.

Patient ID (Gender)	Age (y)	Age of onset (y)	Duration of illness (y)	Vascular history	VaP Family history	Past history of stroke	Onset of illness	MRI	Freezing	Response to levodopa	MDS-UPDRS -III OFF	MDS-UPDRS-III ON	NMSS	MoCA	FAB	Genes harboring candidate genetic variants
1 (M)	73	70	3	Htn	Negative	Positive	Insiduous	Fezekas grade 2	Negative	Positive	50	29	24	19	9	PLA2G6, DNAJC13, UBR4, BSN, NOTCH3, CACNG5
				IHD												
				Dyslipidemia												
2 (F)	60	56	4	Htn	Negative	Positive	Insidious	Fezekas grade 2	Positive	Negative	45	39	39	16	8	LRRK2, CACNA1H, RET, FBLN2, TGM6, STUB1, TRPV4
				IHD												
				Dyslipidemia												
3 (M)	80	75	5	Htn	Negative	Negative	Insidious	Fezekas grade 2	Positive	Negative	41	27	29	22	9	TGM6, BSN, CD36, SCN4A, BRF1, PLA2G6
				IHD												
				Dyslipidemia												
4 (M)	83	79	4	Htn	Negative	Positive	Insidious	Fezekas grade 3	Negative	Negative	34	34	6	21	9	POLG, TNR, UBR4, BSN, AARS1, PRKCH
				Dyslipidemia												
5 (M)	63	59	4	Htn	Negative	Positive	acute	Fezekas grade 3	Negative	Negative	36	34	20	20	9	LRRK2, ATXN3, PLXNA1, SMPD1, KCNK18, KCNC3, PLA2G6, ANGPTL6, ADAMTSL4, LAMB1, CD36, NOTCH3
				Dyslipidemia												

Abbreviations: y: years; VaP: Vascular Parkinsonism; MDS-UPDRS: Movement Disorder Society—Unified Parkinson’s Disease Rating Scale; NMSS: non-motor symptoms scale; MOCA: Montreal cognitive assessment; FAB: Frontal Assessment Battery; MRI :magnetic resonance image ;WMLs : white matter lesions.

No pathogenic mutations, according to ACMG or Clinvar which are intended classifications for monogenic diseases, were found in known PD genes. Variants of uncertain significance, however, were identified in several PD/parkinsonism genes like LRRK2, PLA2G6, EIF4G1, POLG and DNAJC13. Using the voting approach, a total of seven genes were highlighted (Table 2), including monogenic PD genes (LRRK2, PLA2G6), monogenic parkinsonism genes (TGM6, BSN), genes associated with both vascular function and PD (UBR4 and CD36) and monogenic cerebrovascular disease genes (NOTCH3). The whole list of variants in 53 prioritized genes associated with either parkinsonian (39 genes) or cerebrovascular disease (14 genes) categories are provided (Supplementary Table S1).

**TABLE 2:** Variants identified in the 7 prioritized genes by voting approach.

Gene	Chr:Pos:ref>alt	Functional consequence	amino acid change	MAF (1000G/GnomAD)	CADD	dbsnp ID	Brain expression	Patient number	Neurological phenotype in mouse model
LRRK2	chr12:40677735:G>A	Missense	p.R767H	./0.0000323	21.6	rs200861727	y	2	y
	chr12:40740686:A>G	Missense	p.N2081D	0.00978435/0.0108	23.5	rs33995883		5	
PLA2G6	chr22:38528887:G>A	Missense	p.A343V	./0.00003232	20.8	rs1162944680	y	5	y
	chr22:38528888:C>T	Missense	p.A343T	0.00559105/0.0085	20.3	rs11570680		1,3	
TGM6	chr20:2384129:C>T	Missense	p.P359L	—	29.9	rs201024326	y	3	n
	chr20:2397883:C>T	Missense	p.R448W	0.00678914/0.0161	22.7	rs147979536		2	
BSN*	chr3:49699140:G>A	Missense	p.E3288K	—	26.6	.	y	1	y
	chr3:49700621:A>G	Missense	p.H3677R	—	24	.		3	
	chr3:49694041:G>A	Missense	p.R2351Q	0.000399361/0.0004	24.5	rs147218044		4	
NOTCH3	chr19:15299048:G>A	Missense	p.S497L	0.00738818/0.007	25.8	rs114207045	y	1	y
	chr19:15291062:G>A	Missense	p.R1050W	0.000199681/0.00006472	26	rs371525707		5	
UBR4*	chr1:19447881:C>T	Missense	p.V3315M	—	29	rs779342826	y	4	n*
	chr1:19503178:C>A	Missense	p.G894V	0.00299521/0.0011	23.6	rs143130698		1	
CD36	chr7:80292426:G>A	Missense	p.D184N	—	27	rs138897347	y	5	y
	chr7:80301310:T>G	Stopgain	p.L360X	./0.0002	42	rs56381858		3	

Same variant previously reported in disease/functionally characterized	Supporting evidence	References of supporting evidence	Signifiance of amino acid position	Protein function	Human disease (OMIM)	ACMG	Clinvar condition	Clinvar classification
y	Same variant, p.R767H, was previously reported in Taiwanese idiopathic PD patients and was functionally characterized as one of 23 LRRK2 activating mutations by destabilizing the ANK:CH (ankyrin: c-terminal helix) interface. The high kinase activity observed in LRRK2-activating pathogenic mutatants, such as the common PD/parkinsonism risk variant G2019S, is associated with pathological features of PD/parkinsonism	(Kalogeropulou et al., 2022)	Within ankyrin domain (ANK) for protein-protein interactions and required for RAB29-mediated activation; same reported variant in PD	Regulates intracellular vesicle trafficking and organelle maintenance	Parkinson disease 8 AD / 607060	Uncertain significance	.	.
y	Same variant was previously linked to increased PD risk with functionally validated increased LRRK2 activation and enhanced Rab10 phosphorylation. The mutation disrupts the hydrophilic interactions between kinase and LRR domains causing an increase in LRRK2 activity.	(Kalogeropulou et al., 2022; Hui et al., 2018)	Within kinase domain; same reported variant in PD; is an eQTL for LRRK2 expression in brain tissue			Likely benign	Autosomal_dominant_Parkinson_disease_8	Benign/Likely_benign
n	While PLA2G6 parkinsonism cases are mostly bi-allelic, several cases of mono-allelic variant carriers have been reported in atypical parkinsonism. Functional studies on PLA2G6 mouse and fly models suggest that membrane remodeling by PLA2G6 is required for the survival of Dopaminergic neurons and α-Syn stability	(de Oliveira et al., 2021; Ferese et al., 2018; Rabaneda-Lombarte et al., 2022; Gómez de S et al., 2022)	Falls within Ankyrin repeat-containing domain. No available functional characterization but nearest amino acid substituition 2 amino acids away A341T causes PLA2G6-associated neurodegeneration (PLAN) and a complete loss of phospholipase and lysophospholipase activities. Heterozygous p.Ser258Leu, also within ANK repeat domain, is reported in late-onset parkinsonism. The same amino acid is substituted in 3/5 patients; via 2 different variants. the 2nd variant (shared by 2 patients) is an eQTL for NPTXR in brain tissue, a biomarker of synaptic dysfunction associated with multiple neurodegenerative diseases including PD, and reported to be decreased in atypical parkinsonian disorders	Phospholipase; implicated in membrane remodeling specially in mitochondria required for the survival of Dopaminergic neurons and α-Syn stability	Neurodegeneration with brain iron accumulation 2B AR/610217; Parkinson disease 14, autosomal recessive AR/612953	Uncertain significance	Infantile_neuroaxonal_dystrophy	Uncertain_significance
n						Likely benign	PLA2G6-associated_neurodegeneration	Benign/Likely_benign
y	Low frequency monoallelic TGM6 variants have been found to be enriched in PD patients vs. controls, with in-silico predicted deleteriousness, functional validation and co-segregation for some of these variants supporting a role for TGM6 in PD. The same variant, p.P359L has been previously reported as PD-causing variant after WES analyis and co-segregation in a small family affected with autosomal dominant EOPD and has been reported as spinocerebellar ataxia-causing as well.	(Chen et al., 2020; Westenberger et al., 2016)	Within the Transglutaminase-like domain; same reported variant in PD and SCA; very high REVEL score 0.926; evidence of structural damage (Missense3D)	Catalyzes various post-translational modifications in proteins and peptides	Spinocerebellar ataxia 35, AD 613908	Uncertain significance	not_provided	Uncertain_significance
n			This variant has not been previously reported in parkinsonism, however the presence of different rare TGM6 variants in PD patients such as nearby p.V391M which is reported more than once in PD cases (yet is Clinvar benign like this one) could hint that such TGM6 rare variants could contribute to parkinsonism risk, not cause disease through monogenic effect. This variant, like p.V391M, doesn't fall within a characterized domain			Likely benign	Spinocerebellar_ataxia_type_35	Benign/Likely_benign
n	Heterozygous missense mutations in BSN were reported in familial and sporadic PSP-like atypical parkinsonism with co-segregation in familial cases. All the PSP-reported mutations, p.P2855L, p.R3146C, p.G3627V, p.P3866A, fall within disordered regions. one BSN deleterious missense variant, p.R2603Q, was the only prioritized variant after WES analysis in one familial PD patient within a Saudi cohort. BSN mutations have also been associated with epilepsy highlighting its involvement in neurological function.	(Yabe et al., 2018; Yemni et al., 2019; Ye et al., 2023)	Variants p.E3288K, p.H3677R fall under short sequences of compositional bias in disordered regions like PSP-reported mutations and one p.R2351Q in coiled-coil domain .	Plays a role in synaptic vesicles trafficking, is essential for regulating activity at both inhibitor and excitatory glutamatergic synapses. BSN acts in concert with PRKN to control presynaptic autophagy and maintain homeostatic synaptic vesicle turnover	An association between variation in the BSN gene and inflammatory bowel disease/612241 (which has shared genetic etiology with PD and is a risk factor for PD)	Uncertain significance	.	.
n						Uncertain significance	.	.
n						Uncertain significance	.	.
y	This substitution, p.S497L, was reported to significantly alter protein structure (EGFR folding), was only observed in CSVD patients with WMLs and none of the controls in case control study and was recently listed as suggestive for CADASIL and white matter lesions. it was observed, among other rare NOTCH3 variants, in a PD cohort study (139 PD) with an enrichment of 9% of cases harboring such rare variants which were shown to significantly contribute to increased WML burden (double WML in NOTCH3 variant positive vs negative patients)	(Papageorgiou et al., 2024; Vlachakis et al., 2014; Ramirez et al., 2020; Schmidt et al., 2011)	p.S497L and p.R1050W fall within EGF-like- domain repeats; 12th and 27th. the 1st was previously associated with cerebrovascular disease and structurally shown to be deleterious. the 2nd; lies close to disulfide bridge between cys 1055 and 1070, which could be affected by this mutation	Notch signaling pathway displays a significant function in the mature brains beyond neuronal development through regulating neuronal connection, synaptic plasticity, learning, and memory	Cerebral arteriopathy with subcortical infarcts and leukoencephalopathy 1 AD/125310	Likely benign	CADASIL	Benign/Likely_benign
n						Uncertain significance	not_provided	Conflicting_classifications_of_pathogenicity
n	Several heterozygous missense mutations in UBR4 are reported in both sporadic and familial (AD) episodic ataxia. Examples are A2581V, A5042V and R5091H causing AD episodic ataxia, and other mutations such as S958Y and A3263T were reported in sporadic cases presenting with ataxia and brain atrophy, and in adult onset dementia, seizures and white matter abnormailities respectively.	(Conroy et al., 2014; d’Escamard et al., 2024)	Both variants are close to disease-reported variants, none of which (ours nor reported) fall within characterized domains/motifs	E3 ligase that plays a role in the N-end rule pathway and helps degrade aggregation-prone nascent polypeptides		Uncertain significance	not_specified	Uncertain_significance
n						Uncertain significance	not_provided	Likely_benign
n	CD36 has been identified as a risk gene for PD in large PD meta-GWAS study. CD36 variants have been previously associated with both stroke, hypertension and atherosclerosis, as well as PD. In a WES analysis of EOPD dataset, CD36 was prioritized as PD-gene where compound heterozygous (bi-allelic deleterious) variants were found in EOPD cases and in validation datasets (not exclusive to EOPD), multiple PD cases with late-onset PD had mono-allelic deleterious variants and was proposed to act as PD risk gene via an additive model of pathogenicity, implying more severe/early disease onset when the gene has two affected alleles.	(Jansen et al., 2017; Lee et al., 2021; Nalls et al., 2014)	The truncation is before C-terminal region necessary for interaction with other proteins	Multifunctional glycoprotein that acts as a receptor for a broad range of protein ligands like thrombospondin, fibronectin, collagen or amyloid-beta as well as of lipid ligands like fatty acids explaining the pleiotropic effects of the gene and implicating roles in both cerebral ischemia and atherosclerosis, and PD.	Coronary heart disease, susceptibility to/ 610938; Platelet glycoprotein IV deficiency AR/ 608404	Uncertain significance	Platelet-type_bleeding_disorder_10	Conflicting_classifications_of_pathogenicity
n						Pathogenic	Platelet-type_bleeding_disorder_10	Conflicting_classifications_of_pathogenicity

Abbreviations: Chr:Pos:ref>alt: Chromosome:position:reference allele>alternative allele mapped to hg19; MAF: minor allele frequency, CADD: CADD score, brain expression: gene expression in brain tissue (yes/no) according to GTEx, Neurological phenotype in mouse model: (yes/no) abnormal neurological phenotype in genetic mouse model according to MGI https://www.informatics.jax.org/, ACMG: The American College of Medical Genetics and Genomics , OMIM: Online Mendelian Inheritence in Man https://omim.org/, AD: autosomal dominant; AR: autosomal recessive; CSVD: cerebral small vessel disease; WMLs: white matter lesions Genes with * denote genes with high constraint scores (pLi =1 and Z-scores for missense of 3.33 and 8.44, respectively for BSN and UBR4) according to GnomAD v.4.1; https://gnomad.broadinstitute.org/) indicating loss of function and haploinsufficiency are potentially pathogenic; n* nervous system abnormality, lethal due to arrest of vascular remodeling.

Pathway enrichment analysis revealed a significant over-representation of pathways related to collagen and extracellular matrix organization, as well as vascular transport and glycogen catabolism (Supplementary Tables S2, S3).

## Discussion

Parkinsonism can arise from various neurodegenerative conditions, with increasing evidence that mutations in monogenic PD genes may act as susceptibility factors for sporadic forms of parkinsonism (Lesage and Brice, 2012). In VaP, a causal link between clinical strokes and parkinsonism remains elusive. Studies have shown only 10% of cases with striatal or cerebral infarcts, and a third of cases of all types of ischemic strokes develop parkinsonism (Voss and Dorsey, 2010). This possibly suggests that genetic or environmental risk factors, present in this subgroup, are necessary for developing parkinsonism. Relevantly, an association between prior strokes and developing PD was reported by a large retrospective study (Kummer et al., 2019). PD is predominantly sporadic where polygenic and environmental components are assumed to cause disease. These results support a possible interaction between PD/parkinsonism polygenic risk factors and vascular risk factors for developing VaP.

This study investigated genetic risk factors for parkinsonism and cerebrovascular disease in a series of VaP patients, prioritizing variants in 7 relevant genes using a voting approach. These genes include LRRK2, PLA2G6, TGM6, BSN, UBR4, CD36 and NOTCH3 (Table 2).

LRRK2 mutations are a major cause to autosomal dominant inherited late-onset PD as well as sporadic parkinsonism by increasing disease susceptibility (Blauwendraat et al., 2020). In PD recessive genes such as PLA2G6, the prevalence of heterozygous rare variants is significantly higher in familial and sporadic parkinsonism cases than in controls highlighting possible contribution to disease susceptibility, as has been reported for PINK1 and PRKN (Hayashida et al., 2021). The polygenic interaction of such heterozygous mutations in PD genes in PD pathogenesis is also exemplified in familial PD cases with digenic heterozygous mutations in PINK1/DJ1, PINK1/PRKN or PRKN/LRRK2 (Hayashida et al., 2021).

TGM6 and BSN mutations are monogenic causes for late-onset atypical parkinsonism such as spinocerebellar ataxia and progressive supranuclear palsy (PSP)- like syndromes and are associated with PD (Chen et al., 2020; Yabe et al., 2018) PSP-like syndrome with multi-infarct and poor/no L-dopa response has been described as one of the clinical syndromes of VaP (Vizcarra et al., 2015).

CD36 and UBR4 are implicated in both vascular functions and parkinsonism. CD36 was identified as a PD risk gene in PD meta-GWAS study and large-scale PD case control WES study (Jansen et al., 2017) and is also implicated in cerebral ischemia (Lee et al., 2021). UBR4 was identified as a monogenic cause for familial adult-onset episodic ataxia, WMLs and brain atrophy via AD, is associated with PD and early onset dementia, and its role in vascular physiology and functionality was recently unraveled through driving collagen/matrix production necessary for vascular development (Conroy et al., 2014; d’Escamard et al., 2024). Worth mentioning UBR4 and BSN have high constraint scores (GnomAD; https://gnomad.broadinstitute.org/) and a probability of being loss-of-function intolerant (pLI) 1.0, indicating loss of function and haploinsufficiency are potentially pathogenic (Table 2).

NOTCH3 mutations are causative of CADASIL, characterized by recurrent strokes, migraines and not uncommonly late-onset parkinsonism (Rowley, 2013). Characteristic cysteine-altering mutations distributed throughout the 34 epidermal growth factor-like repeats (EGFRs) that comprise the extracellular domain of the NOTCH3 receptor cause the disease with a highly heterogenous clinical phenotype with stroke onset ranging from the fourth to eighth decade (Cho et al., 2022). EGFr 1-6 pathogenic variants are associated with a more severe phenotype compared with EGFr 7–34 22,which was not supported in a recent study reporting severe phenotype in EGFRs 18–34 (Cho et al., 2022). Furthermore, a recent study demonstrated that the disease is not always fully penetrant, by studying the carriers of NOTCH3 cysteine-altering mutations in EGF repeats 1 to 34 in a large exome database (the exome aggregation consortium) reporting a 100-fold higher frequency of CADASIL (Coupland et al., 2018; Papageorgiou et al., 2024).

Recently, some NOTCH3 cysteine-sparing mutations such as (p.R61W, p.R75P, p.D80G, p.R213K, p.L1515P, A1604T) were reported to cause milder vascular disease, referred to as CADASIL-like (Coupland et al., 2018), meeting several but not all clinical criteria for CADASIL, with support from co-segregation genetic analysis from multiple families, structural or functional analysis of the mutations (Coupland et al., 2018). Such cys-sparing mutations may produce an altered Notch signaling output resulting in subtler aspects of vascular disease without causing the entire spectrum of classical CADASIL symptoms Interestingly, a report on 7 cases with a clinical profile of VaP secondary to CADASIL showed that all NOTCH3 mutations were cysteine sparing (Guo et al., 2022). This draws attention to the relevance of NOTCH3 genetic testing in VaP and functional characterization of cys-sparing mutations Furthermore, a link between NOTCH3 genetic variants, PD and WMLs has been previously noted, where PD patients showed an enrichment of rare missense NOTCH3 variants compared to controls, and a doubling of WML burden between NOTCH3 variant positive vs. negative patients with a large effect size for periventricular WMLs and lacunes, as well as a significant negative correlation between WML and global cognition were reported (Ramirez et al., 2020). Interestingly, no association between NOTCH3 genetic variants and early-onset or familial PD was found in a Chinese study (Zeng et al., 2022). The difference in significance of the association between NOTCH3 genetic variants and PD cases not of early age of onset and with WMLs and lacunes, which greatly overlaps with sporadic VaP, but not early onset or familial PD might indicate a possible contribution of NOTCH3 genetic risk variants to VaP or sporadic parkinsonism in general. Relevantly, and in addition to parkinsonism (Vascular parkinsonism) being reported as a late but not rare feature of CADASIL in NOTCH3 genetic variant carriers, parkinsonism has been reported as a novel onset symptom in CADASIL patients with NOTCH3 cys mutations, with similar VaP clinical picture of progressive gait instability, rigidty and WMLs (Wang et al., 2023; Arbeu et al., 2024). Finally, co-occurrence of CADASIL and parkinsonian syndromes (progressive parkinsonism in one case and autopsy-confirmed PD in another), have been reported, further establishing NOTCH3 as a PD and parkinsonism gene (Murray et al., 2017). Taken together, as WES analysis gains broader application, attention has shifted toward the contribution of deleterious mutations in NOTCH3 in PD and parkinsonism, complementing their established association with CADASIL and further underscoring the intersection between parkinsonism and vascular dysfunction (Lesage and Brice, 2012).

In addition to the 7 highlighted genes by voting, other VaP-relevant candidate variants in our prioritized list of vascular and parkinsonian associated genes are reported in supplementary data with their supporting evidence. These genes include: monogenic parkinsonism genes (POLG), monogenic PD genes such as (PARK7, DNAJC13), PD risk genes (SMPD1), PD drug transporter and response gene (SLC22A1), genes harboring the same pathogenic variants previously reported in familial cases of PD (TNR), familial migraine with aura (KCNK18), a risk factor for both strokes and parkinsonism, or genes harboring variants falling in important domains and directly neighboring pathogenic variants previously reported in parkinsonism and familial intracranial aneurysms (PLXNA1 and ANGPTL6).

Enrichment of deleterious variants in collagen and ECM genes, which play a crucial role in blood vessel integrity and elasticity, in our VaP sample supports their reported contribution to both vascular and neurological dysfunction. This significant enrichment was evident in our filtered gene list harboring LoFs and *in silico* predicted deleterious variants, and also evident when using only the most deleterious subset of that list, filtered by REVEL score (Supplementary Tables S2, S3). Genetic susceptibility to periventricular venous collagenosis; i.e., increased collagen deposition in vessels leading to wall-thickening and stenosis, has been proposed as a key factor in WML development, which is common and extensively found in the subcortex in VaP (Lin et al., 2017). Genetic studies consistently implicate different collagen genes, which are core components of the basement membrane in blood vessels, such as in COL4A1/2, COL3A1, COL5A2, in CSVD and stroke through predisposing to arterial wall weakness. Furthermore, collagen genes, COL6A1-6 have been associated with movement disorders such as muscular dystrophies, dystonia, restless leg syndrome, epilepsy and defects in dopaminergic signaling (Gregorio et al., 2022). A new implication for COL22A1, previously associated with muscular dystrophy, in intracranial aneurysms was reported where a heterozygous mutation functionally characterized to impair vessel integrity and increase cerebral hemorrhages in Zebrafish was reported in a patient diagnosed and presenting with VaP, who was later suggested by the authors to be probably misdiagnosed and that the patients’ WMLs were due to adult-onset leukoencephalopathy due to the collagenopathy (Marsili et al., 2023). Collectively, this study highlights the complexity of genetic contributions to VaP through predicted deleterious variants in genes associated with parkinsonism, cerebrovascular disease as well as collagen-related genes. These findings support the notion of a polygenic interplay of risk factors contributing to VaP, rather than it being solely a feature of monogenic leukoencephalopathies due to single mutations in genes such as CSF1R, COL4A1/2, NOTCH3, and COL22A1 as recently proposed (Marsili et al., 2023; Rowley, 2013; Guo et al., 2022). This polygenic model is also more consistent with the sporadic nature of the disease.

Importantly, the presence of variants previously linked to classical Parkinson’s disease (PD) such as LRRK2 p.R767H and p.N2081D, TGM6 p.P359, and TNR p.T166A does not confound the diagnosis of VaP in our cohort. These findings align with current understanding that mutations in monogenic PD-associated genes may serve as risk modifiers for both familial and sporadic forms of parkinsonism (Lesage and Brice, 2012).

Moreover, the identification of variants like NOTCH3 p.S497L, previously reported in CADASIL and PD, and KCNK18 p.F139Wfs*25, associated with familial migraine with aura and proposed as a risk factor for both CADASIL and VaP, reinforces the overlapping phenotypic spectrum among these conditions. Our data support previous observations that parkinsonism may be an initial or co-occurring manifestation in carriers of NOTCH3 variants, especially considering the incomplete penetrance and variable expression of CADASIL.

Given the clinical overlap between VaP, PD, CADASIL and other diseases, and the ongoing debate regarding VaP as a distinct entity, further genetic studies involving larger cohorts are necessary to validate these associations and explore whether these variants represent common genetic risk factors. Future research should also focus on functional characterization of the identified variants and their clinical relevance in the pathogenesis of VaP.

## Data Availability

The datasets presented in this article are not readily available because of ethical and privacy restrictions. Requests to access the datasets should be directed to the corresponding authors.
